# Colour Doppler Imaging of the Ophthalmic Artery During Heart Transplantation

**DOI:** 10.5152/TJAR.2021.21335

**Published:** 2022-10-01

**Authors:** Eda Balcı, Zeliha Aslı Demir, Ayşegül Özgök

**Affiliations:** 1 Department of Anaesthesiology and Reanimation, Ankara City Hospital, Ankara, Turkey

**Keywords:** Cardiovascular and thoracic anaesthesia, cerebral monitoring, colour Doppler imaging, heart transplantation, ophthalmic artery

## Abstract

Colour Doppler imaging of the ophthalmic artery is a non-invasive, fast, and easy access ultrasound technique. Estimation of cerebral perfusion from colour Doppler imaging of the ophthalmic artery is a technique with great potential in this field. In the present case, we monitored blood flow of the ophthalmic artery by colour Doppler ultrasonography during heart transplantation, and we obtained information about the adequacy of the perfusion. Colour Doppler imaging of the ophthalmic artery may be a useful method that can be applied for monitoring cerebral perfusion during heart transplantation and all cardiac operations in order to detect impaired cerebral blood flow.

**Main Points**

- Color Doppler imaging of the ophthalmic artery (OCD) is a non-invasive and easily accessible technique to measure the blood flow velocity of the orbital vessels.
- It can be a useful method for monitoring cerebral perfusion during heart transplantation and all cardiac operations to detect impaired cerebral blood flow.

## Introduction

A complex neurohormonal system, which aims to perfuse tissues properly, aims to compensate for cardiac failure. However, studies show that cerebral blood flow can be reduced in these groups of patients.^1-3^ Besides the existing heart failure, many other factors can affect cerebral blood flow and perfusion during cardiac surgery, such as unrecognized carotid artery stenosis, cardiopulmonary bypass (CPB), non-pulsatile blood flow, circulatory arrest, hypothermia, or embolism.

Colour Doppler imaging of the ophthalmic artery (OCD) is a non-invasive and easily accessible ultrasound measurement used in ophthalmology practice to measure the blood flow velocity of the orbital vessels.^4^ In this report, we would like to report a case in which we used OCD to evaluate cerebral perfusion during heart transplantation surgery.

## Case Presentation

A 54-year-old male patient with inotropic drugs and extracorporeal membrane oxygenation (ECMO) underwent orthotopic heart transplantation. Upon transthoracic echocardiography, ejection fraction was 10% and ventricular wall motions were globally hypokinetic. There was no significant change in carotid and vertebral artery Doppler imaging.

Anaesthesia was induced with midazolam, fentanyl, and ketamine, followed by rocuronium. Maintenance was achieved by sevoflurane inhalation with the guidance of bispectral index (BIS™, Covidien, MN, USA) monitoring, remifentanil infusion, and additional doses of rocuronium. Near-infrared spectroscopy (NIRS, INVOS 5100, Somanetics Corporation, Troy, MI, USA) was measured continuously by sensors placed bilaterally on the patient’s forehead throughout the entire operation, to evaluate cerebral oxygenation. PiCCO (pulse-induced contour cardiac output, Pulsion Medical Systems SE, Germany) monitoring was performed to evaluate cardiac output, stroke volume, and systemic vascular resistance. Mechanical ventilation settings were adjusted so that the tidal volume was 6 mL kg^−1^, positive end-expiratory pressure (PEEP) 3 cmH_2_O, FiO_2_ 50%, and the patient’s PaCO_2_ value was between 35 and 45 mm Hg.

After median sternotomy, an arterial cannula was placed in the femoral artery and a venous cannula was placed in the right atrium. After adequate activated clotting time (ACT) levels, CPB was initiated and the patient’s body temperature was cooled down to 28°C. The target pump flow rate was 2-2.5 L min^−1^m^−2^ during CPB. Mean arterial pressure was kept between 50 and 80 mm Hg. The cardiac allograft was orthotopically implanted using a bicaval anastomotic technique. The total allograft ischemic time was 230 minutes. The haemodynamically stable patient was separated from the CPB pump with noradrenalin (0.4 μg kg^−1^min^−1^), dopamine (5 μg kg^−1^min^−1^), dobutamine (5 μg kg^−1^min^−1^), and isoproterenol (5 μg min^−1^). He received standard heart transplant induction therapy with basiliximab and corticosteroids. After surgery, the patient was admitted to the intensive care unit and extubated 29 hours later uneventfully. He was discharged from the intensive care unit on the third postoperative day in good clinical condition.

### Colour Doppler Imaging of the Ophthalmic Artery

**Figure 1.**
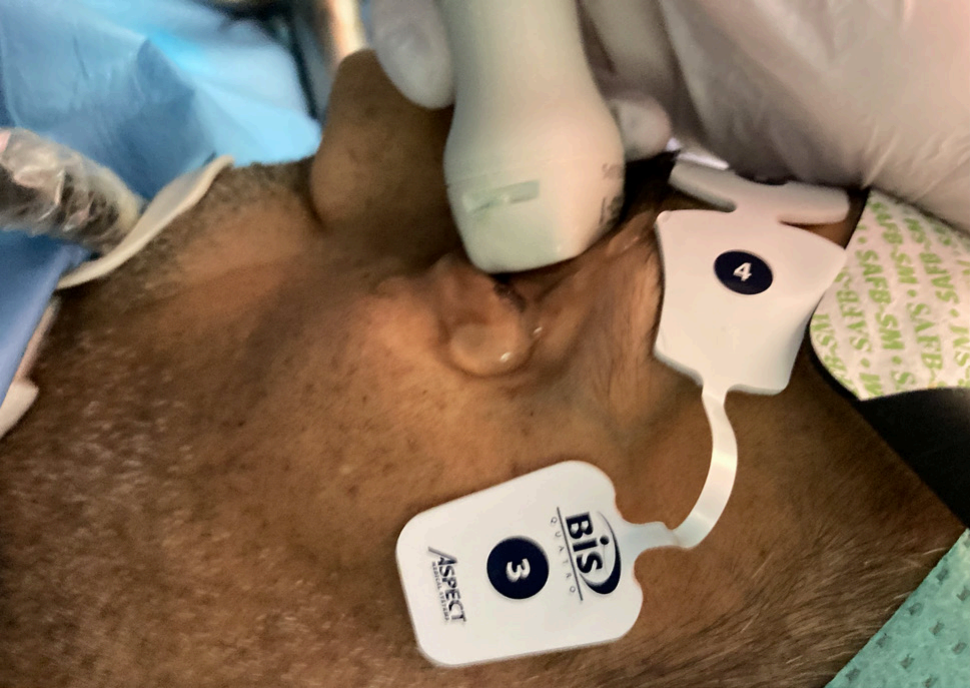
Measurement technique.

**Figure 2.**
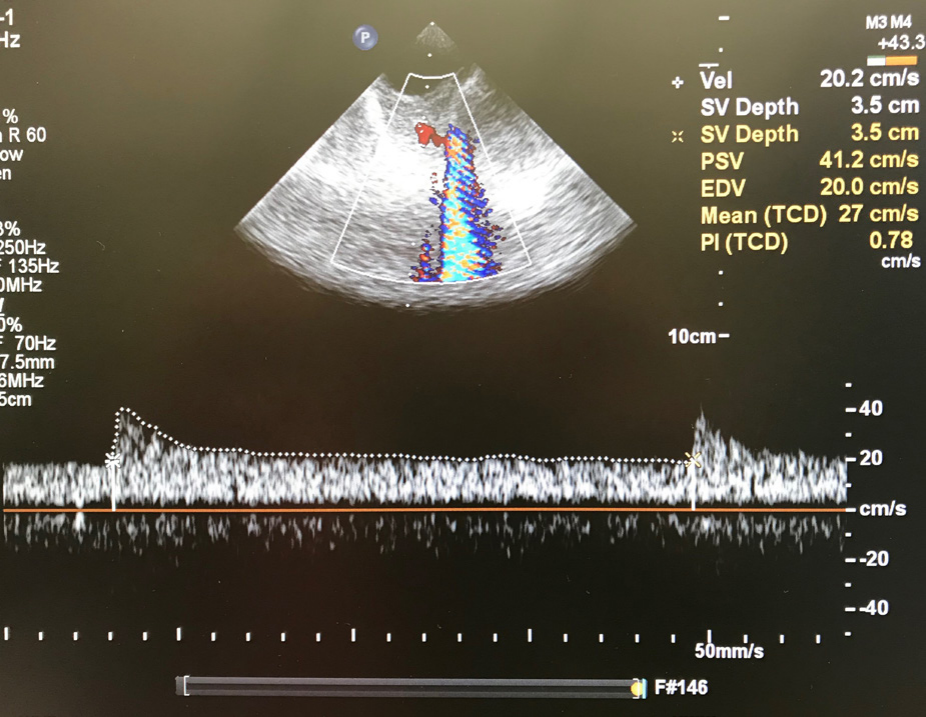
Colour Doppler imaging of the ophthalmic artery, 15 minutes after anaesthesia induction. Ophthalmic artery (OA) mean velocity: 27 cm sec^−1^.

**Figure 3.**
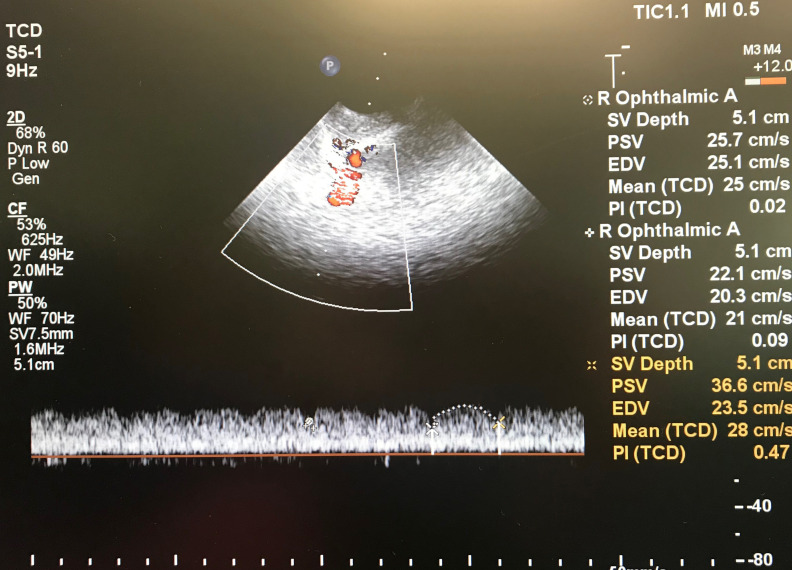
Colour Doppler imaging of the ophthalmic artery, at lowest temperature of cardiopulmonary bypass (28°C). OA mean velocity: 28 cm sec^−1^.

**Figure 4.**
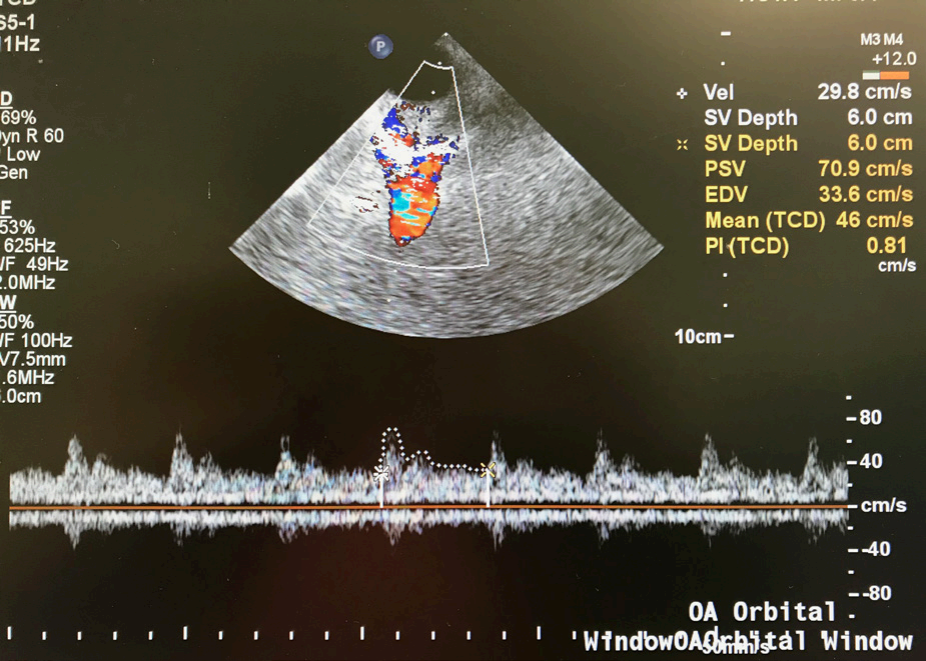
Colour Doppler imaging of the ophthalmic artery, at the end of the operation. OA mean velocity: 46 cm sec^−1^.

The patient was examined in the supine position, with his head inclined at a 30º angle. The eyes were imaged by a 5-1 MHz S5-1 broadband sector array transducer connected to a Phillips EPIQ 7C (*Phillips Medical Systems*). The transducer was used with contact jelly through the closed upper eyelid (Figure 1). The ophthalmic artery blood flow velocity was measured. Systolic velocity, diastolic velocity, mean velocity, pulsatility (PI), and resistance index (RI) were evaluated. The flow velocity was measured at the medial proximal point of the ophthalmic artery using an optimal wall filter preset. Doppler insonation angle was adjusted between 0º and 40º. The first intraoperative ultrasonographic monitoring was started 15 minutes after anaesthesia induction (Figure 2). We performed the second measurement at the lowest temperature during CPB and the third measurement when the sternum was closed after the patient’s surgery was completed (Figure 3 and 4). Measurements were obtained from both eyes and they did not differ from each other, so left eye measurements were recorded. Besides OCD, bilateral NIRS and haemodynamic information were also recorded (Table 1).

Informed consent for publication has been obtained from the patient.

## Discussion

In this report, a patient who was evaluated for intraoperative ophthalmic artery blood flow with colour Doppler imaging and cerebral perfusion with NIRS during heart transplantation operation was presented.

Peripheral vasoconstriction is the compensatory mechanism of low cardiac output. It aims to maintain proper blood perfusion and pressure to the heart and brain. The velocity waveform of OCD provides valuable information about haemodynamic pathophysiology.^4^ The major retrobulbar arteries may be more resistant to vasodilator treatment than the middle cerebral vessel. Studies showed that ophthalmic arterial flow is strictly autoregulated; this provides less sensitivity to changes in overall cerebral perfusion than the middle cerebral artery.^5^ Nevertheless, the small vessel resistance of the ophthalmic artery may have a charge of findings that its peak systolic velocity may more correctly reveal changes in flow during CPB than does either common carotid artery peak velocity or brachiocephalic flow.^6^ In our patient, both ophthalmic artery systolic and diastolic velocities and NIRS values were low in first measurements due to atrial fibrillation rhythm and poor pump function of the ventricle despite ECMO. This was detected by both non-invasive techniques confirming global cerebral hypoperfusion. During CPB, while ophthalmic artery Doppler measurements were low and non-pulsatile, NIRS values were higher than the first measurement. This may be due to hypothermia slowing down cerebral metabolism. Since colour Doppler imaging is the method that evaluates blood flow and NIRS evaluates oxygenation, it is expected to observe this difference during CPB. Despite non-pulsatile flow, the normal level of the cardiac flow (pump flow) during CPB increased cerebral oxygenation. After the donor’s heart was replaced to the patient, the proper pump function of the healthy heart and the inotropic-vasopressor supports increased cardiac output and stroke volume. At the end of the operation, there was an improvement in blood flow and perfusion, and this improvement was observed with both OCD velocities and NIRS values. Kondo et al^7^ suggested that with colour Doppler ultrasonography, orbital blood flow can be easily measured from a perfect acoustic window; the undetectable orbital flow was associated with the occurrence of neurological events in aortic surgery.

Among the cerebral monitoring methods used in cardiac surgery, the evaluation of the middle cerebral artery with transcranial Doppler imaging is a method that has been used for many years.^8^ The most common problem in the visualization of the middle cerebral artery Doppler is that the acoustic window is often closed and the proper imaging is not obtained or it may take a very long time to perform in elderly and comorbid male patients. It has been shown that OCD overcomes some of the technical limitations of transcranial Doppler imaging and they confirmed its anatomic and physiological importance as an indicator of cerebral perfusion during cardiovascular surgery. The ophthalmic artery is easily visualized where it crosses the optic nerve in the orbital cavity.^5^ It is claimed that OCD is a non-invasive and easily applicable measurement technique for the determination of cerebral blood flow during cardiac surgery and that signal differences can signify hypoperfusion of cerebral vessels and thereby indicate inadequate cerebral perfusion.^9^

The fact that the presented case was a heart transplant operation necessitated being careful in terms of using time well. In order not to prolong the ischaemia time of the donor heart, the patient was prepared for surgery quickly. Therefore, the first measurement could only be made after induction of anaesthesia and after all other work had been completed. Starting from the preoperative period, the details of the technique can be revealed with more frequent and bilateral measurements. In addition to all these limitations, the requirement to have basic ultrasonography experience can be added.

We have presented only 1 case here, but this report is important in terms of detailed research of the aforementioned technique and revealing in which cases its use will be beneficial. In cardiac surgery cases, comparing OCD with middle cerebral artery transcranial Doppler technique and evaluating the methods in detail on each patient may guide the future.

## Conclusion

Colour Doppler imaging of the ophthalmic artery may be a useful method that can be applied routinely for monitoring cerebral perfusion during heart transplantation and all cardiac operations to detect impaired cerebral blood flow. Near-infrared spectroscopy is also a monitoring method that is used to evaluate cerebral perfusion. Monitoring brain blood flow and perfusion both with these 2 non-invasive methods may make important contributions to cerebral monitoring in cardiac surgery.

## Figures and Tables

**Table 1.** Intraoperative Variables

	15 Minutes After Anaesthesia Induction	Lowest Temperature of CPB (28°C)	At the End of the operation
OA systolic velocity (cm sec−1)	41	-	70
OA diastolic velocity (cm sec−1)	20	-	33
OA mean velocity (cm sec−1)	27	28	46
NIRS left/right (%)	43/45	58/63	55/58
Bispectral index	40	50	45
Systolic arterial pressure (mm Hg)	83	-	135
Diastolic arterial pressure (mm Hg)	63	-	64
Mean arterial pressure (mm Hg)	69	66	87
Heart rate (beat min−1)	AF 110	-	Pace 100
PaO_2 _(mm Hg)	128	145	138
PaCO_2 _(mm Hg)	32	37	35
SaO_2 _(%)	99	99	99
Cardiac output (L min−1)	3	4.2 (CPB flow)	6.9
Sistemic vascular resistance (dyne sec cm−5)	670	-	980
Stroke volume (mL)	40	-	68
Haemoglobin (g dL^-1^)	8.5	7.6	8.2

OA, ophthalmic artery; NIRS, near-infrared spectroscopy; CPB, cardiopulmonary bypass; AF, atrial fibrillation.
